# A Highly Active Keratinase from *Bacillus* sp. FJ-3-16 for Sustainable Feather Waste Valorization and Eco-Friendly Industrial Applications

**DOI:** 10.3390/biom15101389

**Published:** 2025-09-29

**Authors:** Fei Bian, Hailun He, Gao Chen, Shousong Yue, Yaoxia Zhu, Xiaowei Zhang, Bin-Bin Xie

**Affiliations:** 1 State Key Laboratory of Nutrient Use and Management, Institute of Crop Germplasm Resources, Shandong Academy of Agricultural Sciences, Jinan 250100, China; feifeibian@gmail.com (F.B.);; 2 Jinan Engineering Research Center of Agricultural Microbial Resources Conservation and Biomanufacturing, Shandong Academy of Agricultural Sciences, Jinan 250100, China; 3 Jinan Key Laboratory of Conservation and Utilization of Agricultural Microbial Resources, Shandong Academy of Agricultural Sciences, Jinan 250100, China; 4 School of Life Sciences, Central South University, Changsha 410013, China; 5 State Key Laboratory of Microbial Technology, Microbial Technology Institute, Shandong University, Qingdao 266237, China

**Keywords:** keratinase, keratinous waste, feather hydrolysis, enzymatic dehairing, detergent-compatible enzyme, *Bacillus* sp.

## Abstract

Keratinous biomass, such as feathers, wool, and hair, poses environmental challenges due to its insoluble and recalcitrant nature. In this study, we identified, purified and comprehensively characterized a previously uncharacterized extracellular alkaline keratinase, KerFJ, secreted by *Bacillus* sp. FJ-3-16, with broad industrial application potential. KerFJ was produced at high yield (1800 U/mL) in an optimized cost-effective medium and purified to homogeneity using ion-exchange chromatography. The enzyme exhibited optimal activity at pH 9.5 and 55 °C, with remarkable alkaline and thermal stability, and high tolerance to surfactants, oxidants, and metal ions. Sequence analysis revealed that KerFJ is a member of the serine peptidase S8 family, with a molecular weight of ~27.5 kDa. It efficiently degraded native keratin substrates, achieving 70.3 ± 2.1% feather, 39.7 ± 1.8% wool, and 15.4 ± 1.2% hair degradation, and the resulting feather hydrolysates exhibited strong antioxidant activities. KerFJ also demonstrated excellent compatibility with commercial detergents and enabled effective stain removal from fabrics without damage. Moreover, both laboratory- and pilot-scale trials showed that KerFJ facilitated non-destructive dehairing of sheep, donkey, and pig skins while preserving collagen integrity. These results highlight KerFJ as a robust and multifunctional biocatalyst suitable for keratin waste valorization, eco-friendly leather processing, and detergent formulations.

## 1. Introduction

China is one of the world’s largest producers of livestock and poultry, generating massive amounts of keratin-rich waste each year. China produced approximately 6.2 million metric tons of chicken feathers and 1.9–5.7 million metric tons of pig bristles [1,2], in addition to large quantities of wool, horns, hooves, and hair. These wastes, rich in keratin (91% in feathers [3], 80% in pig bristles [4]), are difficult to degrade due to their tightly packed structure stabilized by disulfide bridges, hydrogen bonds, and ionic bonds. If improperly disposed of, these wastes pose serious environmental and public health threats by contributing to soil and water contamination, pathogenic microorganism spread, and greenhouse gas emissions [5]. Among these keratinous wastes, chicken feathers—with protein content exceeding 85%—represent an abundant and renewable resource that can be converted into high-value products, such as animal feed additives, organic fertilizers, and bioactive peptides [6,7]. However, conventional keratin processing methods, including alkaline hydrolysis, steam pressure treatment, and chemical oxidation, are typically energy-intensive, environmentally harmful, and often result in partial degradation or amino acid loss [8]. In contrast, microbial keratinases offer a selective and eco-friendly alternative for keratin degradation, operating under milder conditions while preserving the nutritional value of the hydrolysates [9]. Most characterized microbial keratinases belong to the subtilisin-like serine protease family (S8) [7]. Although numerous keratinolytic microorganisms have been identified, only a limited number of purified enzymes have demonstrated robust properties required for industrial applications. For example, keratinase from *Bacillus subtilis* FTC02PR1 exhibits optimal activity at 60 °C, retains stability over a broad pH range (6.0–11.0), and shows improved thermal tolerance at 55 °C in the presence of Mn^2+^ [10]. Keratinase from *Bacillus pumilus* AR57 displays optimum activity at pH 9 and 45 °C, with remarkable stability in the presence of DMSO [11]. *Bacillus cereus* IIPK35 keratinase is stable between pH 6.5–10.0 and 35–60 °C, shows optimum activity at pH 9.0 and 55 °C, and tolerates surfactants such as Tween 80 and Triton X-100 [12].

Enzymatic dehairing is emerging as a green alternative to chemical-based leather processing, which traditionally relies on hazardous substances such as lime and sulfides [13]. While some keratinases have shown promise in dehairing goat or sheep hides at the laboratory scale, effective enzymes for more challenging substrates such as pig or donkey skins remain scarce [14,15]. Specifically, pig skins (e.g., local breeds such as Bama xiang pig, *Sus scrofa*) have relatively thick dermis and dense hair roots [16], while donkey hides contain higher levels of fat and oil, both of which complicate enzymatic depilation. Similarly, keratinases show strong potential in detergent formulations by degrading protein-based stains such as blood, sebum, and sweat [17,18,19,20,21]. However, commercial application demands enzymes that can retain activity in the presence of surfactants, oxidants, and under high-pH conditions—performance features that remain underexplored in many candidates [22].

In our previous work, we reported a laboratory-preserved bacterial strain, designated *Bacillus* sp. FJ-3-16, with high keratinase production and preliminary dehairing capability [16]. In the present study, this strain was taxonomically identified for the first time, and its high-performance alkaline keratinase, designated KerFJ, was purified. The enzyme’s biochemical properties were systematically investigated, including its activity profile, stability under various conditions, and compatibility with chemical agents. In addition, KerFJ was evaluated in three industrially relevant applications: feather waste degradation, detergent formulation, and eco-friendly leather dehairing. The resulting feather protein hydrolysate was further analyzed for amino acid composition and antioxidant activity to assess its potential for value-added utilization in animal nutrition or functional materials.

## 2. Materials and Methods

### 2.1. Materials

Chicken feathers and wool were collected from a local slaughterhouse. The materials were cleaned by rinsing with tap water, soaking in 70% ethanol, washing with sterile water, and drying in an oven at 60 °C for 2 h. Dried chicken feathers were ground into fine powder using a laboratory mill to prepare feather meal powder for use in feather meal agar plates. The cleaned feathers, wool, and feather meal powder were subsequently used either in culture media or as natural substrates under sterile conditions.

Human hair was collected from discarded hair at a local barbershop.

Fresh sheep and donkey skins were kindly provided by a local slaughterhouse. A factory-scale dehairing experiment for pig skin, pig heads, and pig trotters was performed at Shandong Lvdu Biotechnology Co., Ltd. (Binzhou, China), as described previously [16].

### 2.2. Medium Preparation

The Minimal medium (g/L) consisted of NaCl 0.5, K_2_HPO_4_ 1.0, KH_2_PO_4_ 0.4, MgCl_2_·7H_2_O 0.1, CaCl_2_ 0.06, adjusted to pH 7.5.

Chicken feather agar plates were prepared by supplementing the minimal medium with 15 g/L feather meal powder and 15 g/L agar, followed by sterilization at 121 °C for 30 min.

Chicken feather liquid medium was prepared by adding 10 g/L raw chicken feathers to the sterilized minimal medium.

Fermentation medium (g/L) contained corn flour 14.1, soy flour 36.2, casein 20.0, CaCl_2_ 1.6, K_2_HPO_4_ 3.0, and KH_2_PO_4_ 1.0, adjusted to pH 8.0. This composition was previously optimized using a combination of one-variable-at-a-time (OVAT), Plackett–Burman (PB), and central composite design (CCD) approaches [16], and was adopted here without further modification.

### 2.3. Isolation of Keratinase-Producing Microorganisms

Soil samples were collected from a slaughterhouse waste disposal site. One gram of soil was suspended in 10 mL of sterile 0.9% NaCl solution and spread onto chicken feather agar plates. After incubation at 37 °C for 48 h, colonies producing clear zones were selected as potential keratinase producers. These colonies were purified and stored at −80 °C for further study.

The keratinase-producing strain FJ-3-16 was previously reported in our earlier work as a laboratory-preserved high keratinase-producing strain with preliminary dehairing capability [16]. Here, the detailed procedure for its isolation and taxonomic identification is described for the first time. Genomic DNA was extracted and the 16S rRNA gene was amplified using primers 27F (5′-AGAGTTTGATCCTGGCTCAG-3′) and 1492R (5′-ACGGCTACCTTGTTACGACTT-3′). PCR amplification was carried out in a 50 µL reaction mixture containing 1 × PCR buffer, 1.5 mM MgCl_2_, 200 µM of each dNTP, 0.5 µM of each primer, 1 U of Taq DNA polymerase (Takara, Japan), and 50 ng of template DNA. The cycling conditions were as follows: initial denaturation at 95 °C for 5 min, followed by 30 cycles of 95 °C for 30 s, 55 °C for 30 s, and 72 °C for 90 s, with a final extension at 72 °C for 10 min. PCR products were purified and subjected to Sanger sequencing (ABI 3730XL platform, BioSune, Shanghai, China). The resulting sequences were compared to entries in the NCBI GenBank database using BLAST (https://blast.ncbi.nlm.nih.gov/Blast.cgi, accessed on 24 July 2025). The identified FJ-3-16 strain was deposited in the China General Microbiological Culture Collection Center (CGMCC) under accession number CGMCC 12086.

### 2.4. Keratinase Production and Purification

For keratinase production in shake-flasks, a single colony of FJ-3-16 strain was inoculated into LB liquid medium and cultured at 30 °C with shaking at 200 rpm for 18 h until reaching the logarithmic growth phase. The seed culture was then inoculated at 6% (*v*/*v*) into the fermentation medium (50 mL in a 500 mL Erlenmeyer flask) and incubated at 37 °C, 250 rpm for 120 h. These fermentation parameters were adopted directly from our previous study [16], where they were determined through statistical optimization.

After cultivation, the culture broth was centrifuged at 12,000 × *g* for 20 min to collect the supernatant. Proteins were precipitated by stepwise ammonium sulfate fractionation, first at 40% saturation to remove non-target proteins and then at 70% saturation to precipitate keratinase, with all precipitation steps performed at 4 °C.

The precipitated proteins were loaded onto a Q-Sepharose™ FF column (1.6 × 20 cm, 30 mL; Cytiva, Marlborough, MA, USA), pre-equilibrated with 50 mM Gly-NaOH buffer (pH 10.0). The flow-through fractions containing keratinase activity were pooled and dialyzed against 50 mM Tris-HCl buffer (pH 7.0) using a dialysis membrane with a 3000 Da molecular weight cutoff (MWCO) at 4 °C, with several buffer changes, then applied to an SP-Sepharose™ FF column (1.6 × 20 cm, 30 mL; Cytiva, Marlborough, MA, USA). The bound proteins were eluted with a linear gradient of 0–0.5 M NaCl in the equilibration buffer (50 mM Tris-HCl buffer, pH 7.0). Fractions exhibiting keratinase activity were analyzed by SDS-PAGE (12.5%) and visualized by Coomassie Brilliant Blue R-250 staining, using an unstained protein molecular weight marker (Pierce™, Cat. No. 26610; Thermo Fisher Scientific, Waltham, MA, USA) as reference. The fraction showing a single protein band was pooled, dialyzed against 50 mM Tris-HCl buffer (pH 8.0), and stored at −20 °C in 20% (*v*/*v*) glycerol for further biochemical characterization and laboratory-scale application potential evaluation.

For the industrial-scale dehairing trial, crude keratinase was produced in a 50 L fermenter using the previously optimized parameters [16], and the culture broth was centrifuged to remove bacterial cells before use, without further purification.

Protein concentration was determined using a BCA Protein Assay Kit (Beyotime Institute of Biotechnology, Shanghai, China), with bovine serum albumin as the standard.

### 2.5. Keratinase Assay

Keratinase activity was measured based on a modified method of Yamamura et al. [23], using keratin powder (TCI, Shanghai, China) as the substrate. One milliliter of appropriately diluted enzyme solution was mixed with 1 mL of 20 g/L keratin suspension and incubated at 45 °C for 1 h. The reaction was terminated by adding 2 mL of 0.4 M trichloroacetic acid (TCA), and the mixture was centrifuged at 12,000× *g* for 10 min.

One milliliter of the resulting supernatant was mixed with 5 mL of 0.4 M Na_2_CO_3_ and 1 mL of Folin–Ciocalteu’s phenol reagent (Sangon Biotech, Shanghai, China), followed by incubation at 40 °C for 20 min. Absorbance was measured at 660 nm.

One unit of keratinase activity was defined as the amount of enzyme that caused an increase of 0.01 in absorbance at 660 nm per minute under the assay conditions, calculated from the linear portion of the reaction time course.

### 2.6. Biochemical Characterization of Purified Keratinase

#### 2.6.1. Effect of pH and Temperature on Keratinase Activity and Stability

The effect of pH on keratinase activity was evaluated across a pH range of 6.0 to 12.0 using the following 50 mM buffer systems: NaH_2_PO_4_–Na_2_HPO_4_ (pH 6.0–7.5), Tris-HCl (pH 7.0–9.0), Glycine–NaOH (pH 9.0–10.5), and Na_2_HPO_4_–NaOH (pH 10.5–12.0). For pH stability assessment, the enzyme was pre-incubated in each buffer at 25 °C for 30 min before activity measurement.

The effect of temperature on enzyme activity was determined by incubating the enzyme-substrate mixture at temperatures ranging from 35 to 70 °C in 50 mM Tris-HCl buffer (pH 8.0). Thermal stability was assessed by pre-incubating the enzyme at 40, 50, and 60 °C in the same buffer, and the residual activity was measured over time. The half-life (t_1_/_2_) of enzyme activity at each temperature was calculated using Equation (1):

ln(A/A_0_) = −0.693/t_1_/_2_ × t (1)

where *A* is the residual activity at time t, and *A*_0_ is the initial activity at t = 0 [24].

#### 2.6.2. Effects of Inhibitors, Metal Ions, Oxidizing/Reducing Agents, Surfactants, and Detergents on Keratinase Activity

To determine the influence of various chemical agents, the enzyme was pre-incubated with individual compounds in 50 mM Tris-HCl buffer (pH 8.0) at 25 °C for 20 min before activity assays. Protease inhibitors included phenylmethylsulfonyl fluoride (PMSF, 1 and 5 mM) and o-phenanthroline (O-P, 1 and 5 mM) (both from Sigma-Aldrich, Merck KGaA, Darmstadt, Germany). Chelators included EDTA (1 and 5 mM) and EGTA (1 and 5 mM). Reducing agents included DTT (5 mM), DTNB (5 mM), and β-mercaptoethanol (5 mM). Oxidizing agents included H_2_O_2_ (3%), NaClO (3%). Surfactants included Triton X-100 (1%), Tween-80 (1%), sodium cholate (1%), saponin (1%) and SDS (1%). Metal ions were tested at 10 mM using chloride salts of Mn^2+^, K^+^, Na^+^, Sr^2+^, Li^+^, Mg^2+^, Ca^2+^, Ba^2+^, Co^2+^, Fe^2+^, Cu^2+^, Ni^2+^, and Zn^2+^. All other reagents were of analytical grade and purchased from domestic suppliers.

Detergent compatibility was evaluated by incubating the purified enzyme with 1% (*v*/*v*) commercial liquid laundry detergents (Liby, Ariel, OMO, Bluemoon, Amway, Kaimi, Supra, Whitecat, Diaopai, and Tide, all purchased from a local supermarket) at 40 °C for 1 h, 6 h, and 12 h, respectively. Prior to use, all detergents were preheated at 100 °C for 10 min to inactivate endogenous proteases. The enzyme activity without detergent treatment was defined as 100%.

#### 2.6.3. Substrate Specificity

The substrate specificity of purified keratinase was assessed by measuring the release of free amino groups from different protein substrates as described by a modified ninhydrin assay [25]. The protein substrates included gelatin, casein, hemoglobin, albumin, azure keratin, and bovine collagen type I (all purchased from Sigma-Aldrich, Merck KGaA, Darmstadt, Germany), as well as laboratory-prepared wool keratin and feather powder which were prepared by grinding cleaned and dried wool or feathers into fine powder as described in Section 2.1.

Five mg of each protein substrate was suspended in 0.5 mL of diluted enzyme solution in 50 mM Tris-HCl buffer (pH 8.0). The mixtures were incubated at 50 °C for 1 h and the reactions were stopped by adding an equal volume of 15% (*w*/*v*) TCA. After centrifugation at 10,000× *g* for 10 min at 4 °C, 0.5 mL of the supernatant was mixed with 1.0 mL of 2% (*w*/*v*) ninhydrin reagent and boiled for 5 min. After cooling, absorbance at 507 nm was measured. One unit of proteolytic activity was defined as the amount of enzyme that liberates 1 µg of amino acid per hour under the assay conditions.

### 2.7. Keratin Waste Degradation and Hydrolysate Analysis

Raw chicken feathers, wool, and human hair (1% *w*/*v*) were thoroughly washed, sterilized with ethanol, and incubated with purified keratinase (2500 U/mL, enzyme: substrate mass ratio ≈ 0.48% *w*/*w*) in 50 mM Tris-HCl buffer (pH 8.0) at 37 °C for 12 h with shaking at 150 rpm. Residual keratin substrates were recovered by filtration using Whatman No.3 filter paper, washed three times with distilled water, and dried at 105 °C to constant weight [26]. The degradation ratio was calculated as:

Biodegradation ratio (%) = (initial weight − final weight)/initial weight × 100.

The hydrolysate supernatant was collected by centrifugation at 8000 pm for 30 min and heated at 98 °C for 5 min to inactivate residual enzyme activity. Soluble protein content in the hydrolysate was measured using a BCA Protein Assay Kit (P0012, Beyotime, China), with bovine serum albumin (BSA) as standard. Amino acid composition was determined using an automatic amino acid analyzer (L-8900, Hitachi, Japan) following acid hydrolysis with 6 mol/L HCl at 110 °C for 24 h under vacuum [27]. Conversion rate of the amino acids was calculated as:

Amino acid conversion ratio (%) = (total amino acid content)/initial weight of the feathers × 100.

### 2.8. Antioxidant Capacity Assays of FPH

The antioxidant capacity of hydrolysate was evaluated using three assays: DPPH (2,2-diphenyl-1-picrylhydrazyl), ABTS [2,2′-azino-bis(3-ethylbenzothiazoline-6-sulfonic acid)], and FRAP (ferric ion reducing antioxidant power).

The DPPH radical scavenging assay was performed based on the method of Wu [28] with slight modifications. Briefly, 20 µL of FPH (100 µg/mL) was added to 100 µL of 0.1 mM DPPH solution (prepared in 95% ethanol) and incubated at room temperature for 1 h. The absorbance was measured at 517 nm using a microplate reader (EnSpire 2300, PerkinElmer, Waltham, MA, USA). Distilled water was used in place of the sample as the blank. Trolox solutions (0.1–1.0 mM) were used to generate a standard curve. Results were expressed as Trolox-Equivalent Antioxidant Capacity (TEAC), calculated from Trolox standard curves and expressed as µmol Trolox equivalents per mg protein (based on the soluble protein concentration of the hydrolysate).

The ABTS and FRAP assays were conducted using commercial kits from Beyotime Biotechnology (Shanghai, China): Total Antioxidant Capacity Assay Kit (ABTS method, Cat. No. S0121) and Total Antioxidant Capacity Assay Kit (FRAP method, Cat. No. S0116). All procedures followed the manufacturers’ instructions. The antioxidant capacity was similarly expressed as TEAC, calculated from Trolox standard curves and expressed as µmol Trolox equivalents per mg protein (based on the soluble protein concentration of the hydrolysate).

### 2.9. Dehairing Assay

Fresh sheep and donkey skins were thoroughly washed, trimmed of fat, and cut into rectangular pieces (3 × 5 cm). Each piece was immersed in 100 mL of purified KerFJ with a final enzyme concentration of ~1.02 × 10^4^ U/mL. Reactions were carried out at 45 °C with shaking at 150 rpm for 8 h. Dehairing progress was monitored every 2 h by gently rubbing the skin surface with tweezers. Control samples were incubated in 100 mL of 50 mM Tris-HCl buffer (pH 8.0) under the same conditions. Each treatment (enzyme and control) was performed in triplicate. After incubation, skins were rinsed with water and assessed for hair removal efficiency, skin surface integrity, and collagen preservation.

A factory-scale dehairing experiment was performed using fresh materials from Bama Xiang pig, including pig skin, head, and hooves, with a total weight of approximately 20 kg. The materials were placed into a large galvanized iron tank (approximately 50–60 L capacity), ensuring that individual pieces were spaced apart to avoid sticking during incubation. Approximately 10 L of crude KerFJ enzyme solution was added to the tank, sufficient to partially immerse all pig parts. The enzyme solution had approximately 3300 U/mL. The tank was equipped with a rotating bottom paddle (estimated 100 rpm) to ensure gentle and uniform mixing. The temperature of the tank was maintained at 45 °C using an electric heating system. The depilation process lasted for 7 h, during which hair removal progress was visually inspected every 2 h.

### 2.10. Destaining Assay

To evaluate the stain removal efficiency of KerFJ, five common household stains—ginger powder, tomato sauce, thick chili paste, sweet soybean paste, and whole blood—were applied to square cotton cloth pieces (5 cm × 5 cm), which were then dried at 50 °C for 1 h.

For destaining, each stained piece was immersed in 20 mL of 1% (*v*/*v*) commercial liquid detergent (Ariel), with or without purified KerFJ (final concentration: 4087 U/mL), and incubated at 40 °C for 30 min with gentle shaking (100 rpm). After washing, cloths were rinsed thoroughly with tap water and air-dried. Three groups were tested in parallel: (i) tap water only (blank control), (ii) detergent without enzyme (detergent control), and (iii) detergent supplemented with KerFJ. All treatments were conducted in triplicate.

To quantitatively assess destaining performance, reflectance measurements were performed on the washed fabrics using a UV–Vis spectrophotometer (UV/VIS-2202, Hitachi, Japan) equipped with an integrating sphere in reflectance mode across a wavelength range of 300–800 nm (data interval 1 nm, spectral bandwidth 2 nm). A clean, unstained cotton swatch was used as the 100% reflectance reference. The average reflectance between 450–750 nm was calculated and used as an indicator of fabric brightness. Reflectance improvement was reported as ΔR (%) = $\bar{R}$(KerFJ) − $\bar{R}$(Ariel).

### 2.11. Scanning Electron Microscope Observation

Scanning electron microscopy (SEM; Quanta 250 FEG, FEI, Hillsboro, OR, USA) was employed to examine the microstructural effects of enzymatic treatment on both depilated pig skin and destained cotton cloth. Samples were dried and sputter-coated with gold for 3 min using a Cressington 108 Auto Sputter Coater (Cressington Scientific Instruments, Watford, UK), followed by imaging with a scanning electron microscope at an accelerating voltage of 10–15 kV.

### 2.12. Gene Sequence Determination and Bioinformatics Analysis

The whole genome of *Bacillus* sp. FJ-3-16 was sequenced in this study using the Illumina platform, assembled with SPAdes, and annotated with the NCBI Prokaryotic Genome Annotation Pipeline (PGAP).

The purified keratinase was resolved by SDS-PAGE and transferred onto a PVDF membrane. The N-terminal amino acid sequence was determined by Edman degradation using an 473A gas-phase sequencer (Applied Biosystems, Foster City, CA, USA).

The 15-residue N-terminal sequence was used as a query to search the predicted proteome of *Bacillus* sp. FJ-3-16 generated from the genome sequence obtained in this study using BLAST (version 2.12.0+). A matching sequence (14/15 residues) was identified as KerFJ, and its corresponding gene sequence was obtained and deposited in GenBank under accession no. MZ516797.1.

Multiple sequence alignment of keratinases was performed using MUSCLE v3.8.31 [29]. A three-dimensional structural model of mature KerFJ was generated with SWISS-MODEL (http://www.expasy.org/swissmod/, accessed on 24 July 2025), using the PDB structure 1SUB (chain A) as a template. The model quality was validated using SWISS-MODEL’s built-in assessment tools. The predicted structure was visualized and analyzed using PyMOL version 2.4.0a0 (http://www.pymol.org, accessed on 24 July 2025).

### 2.13. Sequence Deposition

The 16S rRNA gene sequence of *Bacillus* sp. FJ-3-16 was deposited in NCBI nucleotide database under accession number of MW677222.1. The full-length kerFJ gene sequence was submitted to GenBank under accession number MZ516797.1.

## 3. Results

### 3.1. Identification of Strain FJ-3-16

The keratinase-producing strain FJ-3-16, previously reported as a laboratory-preserved high keratinase producer with preliminary dehairing capability [16], was taxonomically identified in detail in the present study. The 16S rRNA gene sequence was amplified and deposited in the NCBI nucleotide database under accession number MW677222.1. BLASTN analysis revealed 99.87% identity and 100% query coverage to members of the *Bacillus* genus, and the strain was accordingly identified as *Bacillus* sp. FJ-3-16. The strain has been deposited in the China General Microbiological Culture Collection Center (CGMCC) under accession number CGMCC 12086.

### 3.2. Production of Keratinase KerFJ

*Bacillus* sp. FJ-3-16 exhibited substantial keratinase activity (80 U/mL) after 48 h of cultivation in chicken feather liquid medium. The keratinase production conditions were previously optimized using OVAT, PB, and CCD approaches [16] resulting in an optimal shake-flask medium containing corn flour 14.1 g/L, soy flour 36.2 g/L, casein 20.0 g/L, CaCl_2_ 1.6 g/L, K_2_HPO_4_ 3.0 g/L, KH_2_PO_4_ 1.0 g/L, with an initial pH of 8.0. Under these optimized shake-flask conditions, the previous study reported an activity of 166.08 U/mL at 48 h, where one unit of keratinase activity was defined as the amount of enzyme causing an increase of 0.1 in absorbance at 660 nm per minute.

**Figure 1:**
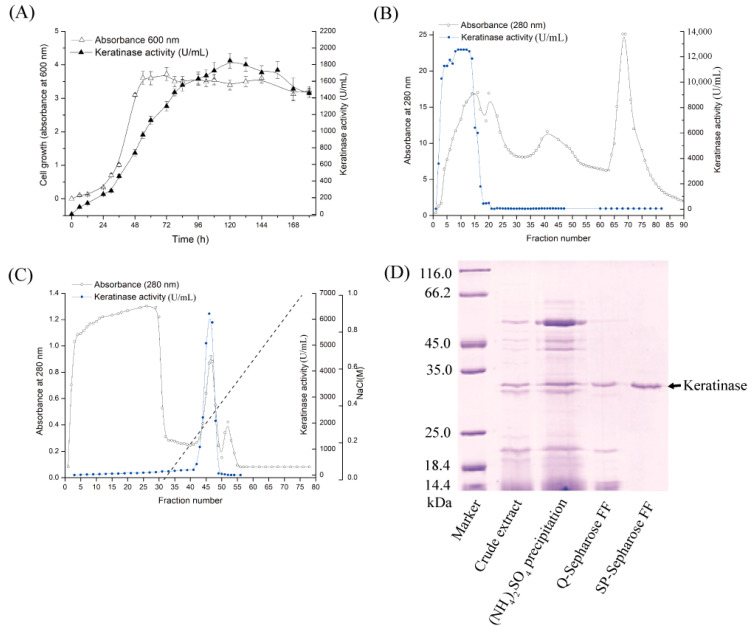
Production and purification of keratinase KerFJ from *Bacillus* sp. FJ-3-16. (**A**) Time course of bacterial growth (Δ) and keratinase production (▲) in the optimized fermentation medium. (**B**) Anion-exchange chromatography of the crude extract using Q-Sepharose™ FF column equilibrated with 50 mM Gly-NaOH buffer (pH 10.0); elution was monitored by absorbance at 280 nm. KerFJ activity was detected in the flow-through fractions, while bound proteins were not eluted with a salt gradient. (**C**) Cation-exchange chromatography using SP-Sepharose™ FF column equilibrated with 50 mM Tris-HCl buffer (pH 7.0); the flow-through fractions from the Q-Sepharose™ FF column were loaded, and the bound proteins on the SP-Sepharose™ FF column were eluted with a linear gradient of 0–0.5 M NaCl in the equilibration buffer; elution monitored at 280 nm. (**D**) SDS-PAGE analysis of purification steps. Lane 1: protein marker (14.4–116.0 kDa); Lane 2: crude extract; Lane 3: (NH_4_)_2_SO_4_-precipitated and dialyzed sample (40–70% saturation); Lane 4: Q-Sepharose™ FF-purified fraction; Lane 5: final purified KerFJ showing a single ~27 kDa band.

In the present work, the above optimized medium and operating parameters were adopted, but the cultivation period was extended to 120 h to maximize enzyme yield for downstream applications. Under these conditions, the activity reached 1800 U/mL at 37 °C (Figure 1A), where one unit was defined as the amount of enzyme causing an increase of 0.01 in absorbance at 660 nm per minute. The observed difference in absolute activity values compared to the previous report is therefore attributable, in part, to the different unit definitions, in addition to the extended cultivation time. Compared with the baseline activity of 80 U/mL in chicken feather liquid medium, this represented a 22.5-fold improvement. These results demonstrate the effectiveness and cost-efficiency of the optimized fermentation strategy, supporting its potential for industrial-scale applications.

### 3.3. Purification of Keratinase KerFJ

The extracellular keratinase, designated KerFJ, was purified through a simplified two-step ion-exchange chromatography protocol involving anion-exchange (Figure 1B) and cation-exchange chromatography (Figure 1C). During Q-Sepharose™ FF chromatography, KerFJ activity was detected in the flow-through fractions, as the enzyme did not bind under the chosen buffer conditions. In contrast, in the subsequent SP-Sepharose™ FF step, the enzyme bound to the column and was eluted with a linear gradient of 0–0.5 M NaCl in 50 mM Tris-HCl buffer (pH 7.0). SDS-PAGE analysis revealed a single protein band with an estimated molecular weight of ~27 kDa (Figure 1D), indicating electrophoretic homogeneity. As summarized in Table 1, the purified enzyme exhibited a specific activity of 53,000 U·mg^−1^, with a final yield of 25% and a 50-fold purification.

### 3.4. Enzymatic Properties: Effects of pH, Temperature, and Substrate Specificity

**Figure 2:**
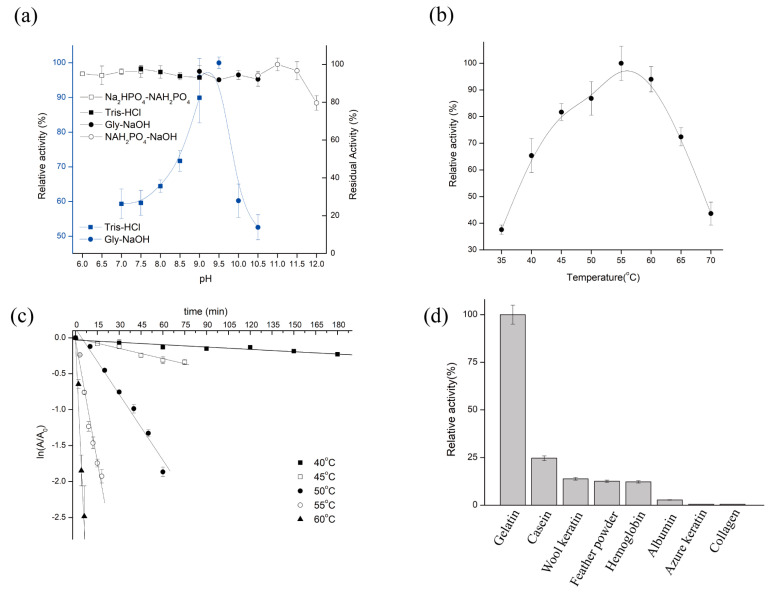
Biochemical properties and substrate specificity of purified keratinase KerFJ. (**a**) Effect of pH on KerFJ activity (blue line) and pH stability (black line). Enzymatic activity was measured at 37 °C across the pH range of 6.0–12.0. pH stability was determined by pre-incubating the enzyme at 25 °C for 30 min in buffers of corresponding pH, followed by standard activity assays. (**b**) Effect of temperature (35–70 °C) on KerFJ activity measured at pH 8.0. (**c**) Thermal stability of KerFJ evaluated by residual activity following pre-incubation at 40 °C, 50 °C, and 60 °C over different time periods. (**d**) Substrate specificity of KerFJ toward various protein substrates. Activity toward gelatin was set as 100%. Error bars represent standard deviation (*n* = 3). Statistical significance was evaluated by one-way ANOVA with Tukey’s post hoc test; *p* < 0.05 was considered statistically significant.

The catalytic activity of purified KerFJ was strongly influenced by pH and temperature. The enzyme exhibited maximum activity at pH 9.5 and retained over 50% of its peak activity across a broad range from pH 7.0 to 10.5 (Figure 2a). Notably, KerFJ demonstrated excellent alkaline stability: following 30 min pre-incubation at 25 °C, the enzyme retained nearly 100% of its initial activity between pH 6.0 and 11.5, and over 80% even at pH 12.0. These results suggest that KerFJ is highly stable under alkaline conditions, which is advantageous for industrial processes such as detergent formulation and leather processing.

KerFJ exhibited optimal activity at 55 °C and maintained over 85% of its peak activity in the temperature range of 55–60 °C (Figure 2b). However, the enzyme’s thermal stability decreased with increasing temperature. As shown in Figure 2c, the half-life (t_1_/_2_) of KerFJ was 10.6 h at 40 °C and 2.4 h at 45 °C. While the enzyme is moderately heat-stable, its high catalytic activity at elevated temperatures supports its use in short-duration high-temperature applications.

In addition to pH and temperature effects, the substrate specificity profile revealed that KerFJ exhibited highest activity toward gelatin (set as 100% ± 2.0%; 12,955 ± 260 U/mg), followed by casein (24.7 ± 1.2%; 3200 ± 155 U/mg), wool keratin (13.9 ± 0.9%; 1801 ± 117 U/mg), feather powder (12.6 ± 1.0%, 1632 ± 130 U/mg), hemoglobin (12.3 ± 0.8%; 1593 ± 104 U/mg), and albumin (2.81 ± 0.3%; 364 ± 39 U/mg). No detectable activity was observed against azure keratin or collagen (Figure 2d). This suggests that KerFJ preferentially hydrolyzes loosely structured or partially unfolded protein substrates. Although its specific activity toward native keratinous substrates such as feather powder and wool keratin was relatively lower, the enzyme retained measurable activity, indicating potential for pretreatment-assisted keratin hydrolysis or synergistic formulations with unfolding agents.

### 3.5. Influence of Various Chemical Agents and Metal Ions on Keratinase Activity

The effects of various chemical agents on KerFJ activity are summarized in Table 2.

Inhibitor assays indicated that KerFJ activity involves a catalytic serine residue and may also require metal ions. The enzyme was partially inhibited by 1 mM PMSF (85.21 ± 3.51%) and strongly inhibited at 5 mM (26.88 ± 1.29%), indicating the involvement of a serine residue in catalysis. The metalloprotease inhibitor O-P had a mild inhibitory effect (91.45 ± 2.91% at 1 mM; 88.93 ± 3.16% at 5 mM). Stronger inhibition was observed with the metal-chelating agents EDTA (26.88 ± 1.65% at 1 mM; 12.87 ± 2.26% at 5 mM) and EGTA (15.81 ± 1.57% at 1 mM; 13.51 ± 1.77% at 5 mM), suggesting that metal ions are also essential for enzymatic activity.

KerFJ showed high tolerance to reducing and oxidizing agents. It retained full or enhanced activity in the presence of 5 mM DTT (100.28 ± 4.32%), β-mercaptoethanol (112.26 ± 2.22%), and DTNB (103.38 ± 4.10%). Interestingly, 3% H_2_O_2_ slightly increased activity (102.96 ± 0.93%), and although 3% NaClO caused moderate inhibition (76.34 ± 3.12%), the residual activity remained acceptable for potential oxidative environments.

With respect to surfactants, KerFJ showed excellent compatibility with several non-ionic and mild ionic surfactants. Notably, saponin (203.81 ± 7.49%), Triton X-100 (140.03 ± 6.55%), and Tween-80 (132.56 ± 3.44%) significantly enhanced enzymatic activity, suggesting potential for detergent formulation. Sodium cholate had a mild inhibitory effect (87.29 ± 3.10%), while SDS drastically reduced activity (30.81 ± 1.12%), consistent with the denaturing effects of strong anionic detergents.

Metal ion effects revealed that Mn^2+^ had the most significant activating effect. K^+^, Na^+^, Li^+^, and Sr^2+^ also enhanced KerFJ activity to varying degrees. Mg^2+^, Ca^2+^, and Ba^2+^ had negligible influence, maintaining near-control activity levels. In contrast, Co^2+^, Fe^2+^, Cu^2+^, Ni^2+^, and especially Zn^2+^ strongly inhibited activity, indicating potential binding interference with the active site or protein conformation (Table 3).

### 3.6. Stability and Compatibility of KerFJ with Commercial Detergents

The compatibility of KerFJ with commercially available laundry detergents was evaluated, and the results are summarized in Table 4. After 1 h of incubation, KerFJ activity was significantly enhanced in detergents such as Liby (126.86 ± 4.44%), Ariel (118.39 ± 0.77%), and OMO (112.99 ± 3.91%), indicating that certain detergent components may facilitate substrate accessibility or stabilize the enzyme conformation. Moderate enhancement or stability was also observed with Bluemoon (112.87 ± 1.05%), Amway (110.38 ± 1.57%), and Kaimi (104.55 ± 1.13%), demonstrating KerFJ’s broad compatibility across diverse formulations.

In contrast, enzyme activity was reduced by Supra (97.28 ± 1.52%), Whitecat (88.99 ± 3.01%), Diaopai (49.06 ± 0.30%), and Tide (34.91 ± 1.84%). Notably, the inhibitory effects of Whitecat, Diaopai, and Tide became more pronounced over time, with residual activity dropping to <15% after 12 h, suggesting potential denaturation or irreversible inhibition in the presence of specific additives.

Despite these exceptions, KerFJ retained > 100% of its original activity after 12 h in Liby, Ariel, and OMO, underscoring its excellent long-term stability and compatibility with mainstream detergent products. These properties support the potential utility of KerFJ as a robust additive enzyme in laundry formulations, especially for applications targeting keratinous stains. It is worth noting that even in the absence of detergent, KerFJ exhibited a slight decline in stability, with residual activity decreasing from 100% to 85% after 12 h at 25 °C. Therefore, part of the activity loss observed in certain detergent formulations may be attributable to intrinsic thermal or temporal inactivation rather than detergent-induced denaturation alone.

### 3.7. Keratin Waste Degradation and Bioactive Potential of Feather Protein Hydrolysate

To assess the keratin-degrading efficiency of KerFJ, three native keratin substrates—chicken feather, wool, and human hair—were incubated with purified enzyme (2500 U/mL) at 37 °C for 12 h. The biodegradation ratios were calculated based on dry weight loss, yielding 70.3 ± 2.1% degradation for feather, 39.7 ± 1.8% for wool, and 15.4 ± 1.2% for human hair. These results indicate that KerFJ is particularly effective in degrading feather keratin, while showing moderate activity on wool and limited activity on human hair.

**Figure 3:**
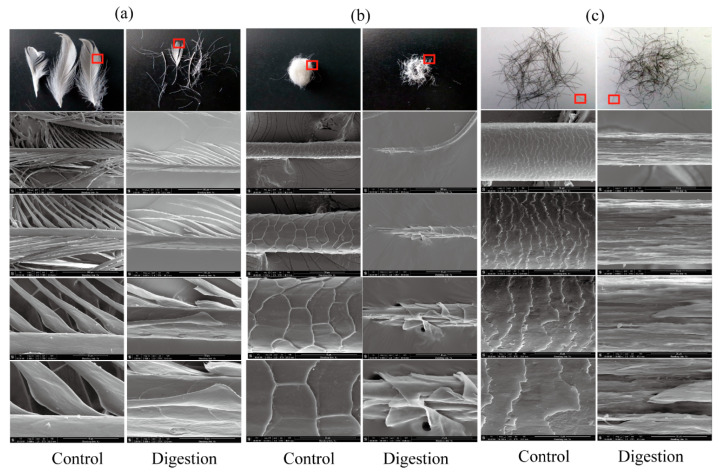
Visual and SEM observations of the biodegradation performance of purified keratinase KerFJ on keratin substrates. (**a**) Chicken feather before and after enzymatic treatment, showing fragmentation of the rachis, barbs, and barbules, with surface roughness and cavity formation. Images (from top to bottom) include visual observation and SEM micrographs at ×500, ×1000, ×2500, and ×5000 magnification. (**b**) Wool fibers after treatment, displaying disrupted cuticle layers and a fragmented cortex. Images (from top to bottom) include visual observation and SEM micrographs at ×1000, ×2500, ×5000, and ×10,000 magnification. (**c**) Human hair before and after degradation, with clear cuticle erosion and surface damage, while the cortex remained largely intact. Images (from top to bottom) include visual observation and SEM micrographs at ×2500, ×5000, ×10,000, and ×20,000 magnification.

Morphological changes during degradation were examined by SEM at different magnifications. Untreated chicken feather showed intact rachis and orderly barbules, whereas enzymatically treated feather displayed significant surface erosion, fragmentation of barbs, and cavities formed by barbule detachment (×500, ×1000, ×2500, and ×5000; Figure 3a). Wool exhibited partial degradation, with separation and disintegration of the outer cuticle layer and damage to the cortical structure (×1000, ×2500, ×5000, and ×10,000; Figure 3b). In human hair, the cuticle was partially removed, exposing the cortex, but the inner structure remained relatively intact (×2500, ×5000, ×10,000, and ×20,000; Figure 3c).

Under the experimental conditions (1% *w*/*v* feather substrate, 2500 U/mL KerFJ), the resulting feather protein hydrolysate contained 2.69 mg/mL soluble protein, corresponding to a protein recovery rate of approximately 38.4% based on the degraded substrate, indicating extensive conversion of insoluble keratin into soluble peptides.

The amino acid profile, summarized in Table 5, revealed a relatively higher abundance of phenylalanine, valine, methionine, alanine, and lysine, which are valuable for nutritional and functional applications. Based on the total amino acid content, the amino acid conversion ratio was estimated to be 63.5%, calculated as the total amino acid content relative to the initial feather weight, suggesting efficient hydrolysis of feather keratin into free amino acids under the applied enzymatic conditions.

Furthermore, the antioxidant potential of the hydrolysate was evaluated. The feather protein hydrolysate exhibited measurable antioxidant activity, with a DPPH scavenging activity of 41.47 ± 0.11 µmol TE/mg protein, and ABTS radical scavenging activity of 95.50 ± 2.57 µmol TE/mg protein, and FRAP (ferric reducing antioxidant power) of 0.083 ± 0.002 µmol TE/mg protein.

These findings collectively demonstrate that KerFJ not only disrupts the rigid structure of keratinous waste materials but also generates bioactive peptides with antioxidant properties, suggesting potential applications in feed formulation or functional materials.

### 3.8. Dehairing of Animal Skin

Dehairing represents a major industrial application of keratinases, particularly in eco-friendly leather processing and hygienic meat production. Previously, crude enzyme from *Bacillus* sp. FJ-3-16 was shown to effectively depilate difficult-to-process pig skins in the context of food processing [16]. In this study, we extended the evaluation to purified KerFJ and different animal skins. KerFJ offered a promising enzymatic alternative for efficient and non-destructive depilation of sheep and donkey hides, expanding its potential beyond porcine substrates.

**Figure 4:**
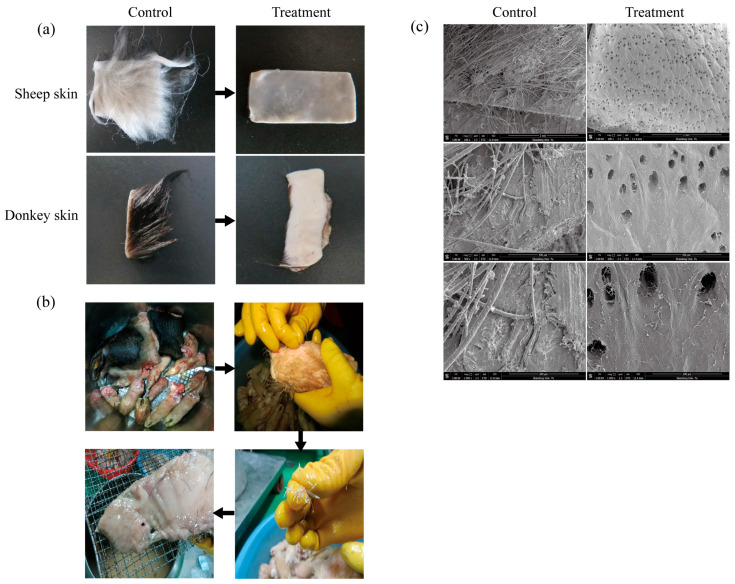
Visual and SEM evaluation of the dehairing performance of keratinase KerFJ. (**a**) Complete hair removal from sheep and donkey skins following treatment with ~ 1.02 × 10^4^ U/mL of purified KerFJ at 45 °C for 8 h, showing smooth surfaces without visible damage. (**b**) Pilot-scale application of crude KerFJ to 20 kg of pig skin, head, and hooves (Bama Xiang pig) enabled efficient depilation by gentle hand-pulling after 6–7 h. (**c**) SEM analysis of depilated pig skin revealed cleanly emptied hair follicles and well-preserved collagen structure, confirming the non-destructive action of KerFJ and the absence of collagenase activity. Images (from top to bottom) are shown at ×100, ×500, and ×1000 magnification.

In laboratory trials, purified KerFJ (~1.02 × 10^4^ U/mL, 45 °C, 8 h) effectively removed hair from sheep and donkey skins without causing visible damage to the skin surface (Figure 4a). Visual inspection revealed complete hair removal, clean follicles, intact skin texture, and no residual stubble. No visible alterations were observed in the collagen layer, suggesting selective degradation of keratin without obvious compromise of the skin’s structural integrity.

Previously published studies demonstrated that crude enzyme from *Bacillus* sp. FJ-3-16 is effective for depilating difficult-to-process pig skins, such as pig heads and trotters [16]. To further evaluate the industrial applicability of KerFJ, a factory-scale dehairing experiment was carried out using fresh materials from Bama Xiang pig, including pig skin, head, and hooves (total weight approximately 20 kg). The materials were incubated in a galvanized iron tank containing ~10 L of crude KerFJ solution at 45 °C, with gentle agitation provided by a rotating bottom paddle. The depilation process lasted for 7 h, during which hair loosening was monitored every 2 h. By 6 h, pig hair had visibly loosened and could be manually removed with minimal force (Figure 4b). After 7 h, complete or near-complete depilation was achieved, and the pig skin, head, and hooves appeared clean and undamaged upon rinsing, indicating the effectiveness of KerFJ for non-destructive hair removal at a pilot/industrial scale. Importantly, the residual enzyme activity in the crude solution remained above 80% after the first depilation cycle, enabling its reuse for a second cycle. In the second run, complete depilation was achieved within 10 h. This demonstrates that crude KerFJ not only performs well under industrial conditions but also retains sufficient activity for multiple uses, thereby significantly reducing operational costs and enhancing the economic feasibility of enzymatic depilation for large-scale applications.

To verify structural preservation, SEM was conducted on the enzymatically treated pig skin. The micrographs confirmed that hair follicles were cleanly emptied without residual hair shafts, and the collagen fibers remained well-organized and intact, indicating the absence of collagenase activity in KerFJ (Figure 4c).

Together, these findings demonstrate that KerFJ, whether in purified or crude form, efficiently and selectively degrades keratin while preserving the structural and functional integrity of the hide. These characteristics make KerFJ a promising candidate for environmentally friendly leather processing, hygienic meat production, and medical skin preparation applications.

### 3.9. Destaining of Stained Cloth

To assess the utility of KerFJ as a detergent additive, its ability to remove common household stains was tested. Cloth pieces were stained with ginger powder, chili paste, soybean paste, tomato sauce, and blood, then washed with 1% Ariel detergent alone or in combination with purified KerFJ.

**Figure 5:**
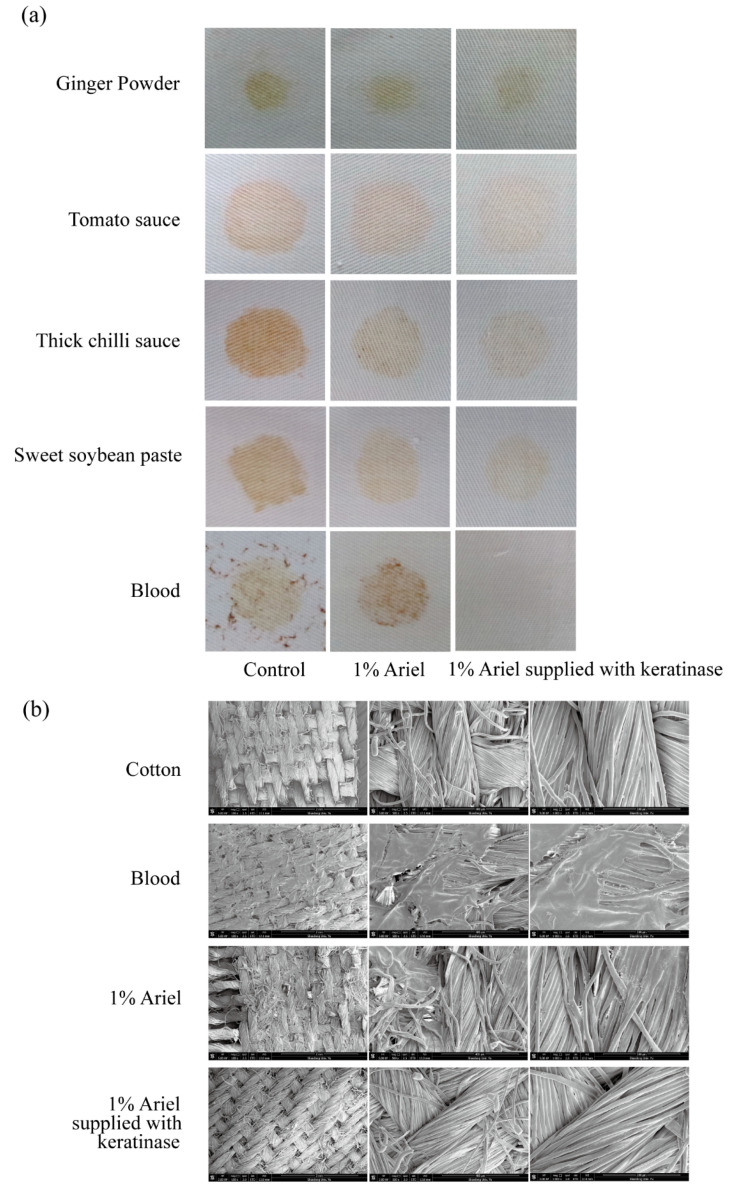
Evaluation of purified keratinase KerFJ as a detergent additive for stain removal. (**a**) Visual comparison of stained cotton fabrics after washing with 1% Ariel detergent alone or supplemented with KerFJ. Enhanced removal was observed for multiple common household stains, particularly blood. (**b**) SEM images of cotton fibers at ×100, ×500, and ×1000 magnification (from left to right) show complete removal of blood residues in the KerFJ-treated group, with surface morphology comparable to that of unstained controls.

Visible improvement in stain removal was observed for all five stains upon addition of KerFJ, with the most notable enhancement seen in the removal of blood stains (Figure 5a). Quantitative analysis of fabric brightness revealed significantly higher reflectance values in the enzyme-supplemented group compared to the detergent-only and tap water controls (Table 6). Specifically, KerFJ addition improved reflectance by 8.4–16.2% across different stain types, with the highest gain observed for blood (+16.2%), followed by tomato sauce (+11.1%) and chili paste (+10.7%). These differences were statistically significant (*p* < 0.05), confirming the additive cleaning power of KerFJ over detergent alone.

SEM imaging further validated the stain removal efficacy of KerFJ on blood-stained fabrics, which showed the highest improvement in reflectance. In the detergent-only group, residual blood-derived deposits remained entrapped within the cotton fiber matrix. In contrast, the KerFJ-treated fibers appeared smooth and clean, comparable to those of unstained controls (Figure 5b), suggesting efficient removal of proteinaceous stains at the microscopic level.

### 3.10. Determination and Analysis of the KerFJ Gene Sequence

The N-terminal amino acid sequence of the purified KerFJ was determined by Edman degradation and identified as AQSVPYGVSQIKAPA. Using this sequence as a query, the corresponding gene (*kerFJ*) was identified from the genome sequence of strain FJ-3-16 obtained in this study. The full-length *kerFJ* gene (GenBank accession no. MZ516797) consists of 1149 bp, encoding a polypeptide of 382 amino acid residues. The identified N-terminal sequence corresponded to residues 108–122, indicating that the mature form of KerFJ comprises residues 108–382. The calculated molecular weight of this region (~27.5 kDa) was consistent with the experimentally determined molecular weight (~27 kDa) observed by SDS-PAGE, confirming the predicted signal peptide cleavage site.

Classification of KerFJ using the MEROPS peptidase database assigned it to the S8 serine peptidase family (MEROPS ID: S08.034), with the closest matches being subtilisin BPN′ from *B. methylotrophicus* and *Bacillus* sp. L010 (both 97.9% identity). This classification is consistent with biochemical evidence, where enzyme activity was partially inhibited by the serine protease inhibitor PMSF.

**Figure 6:**
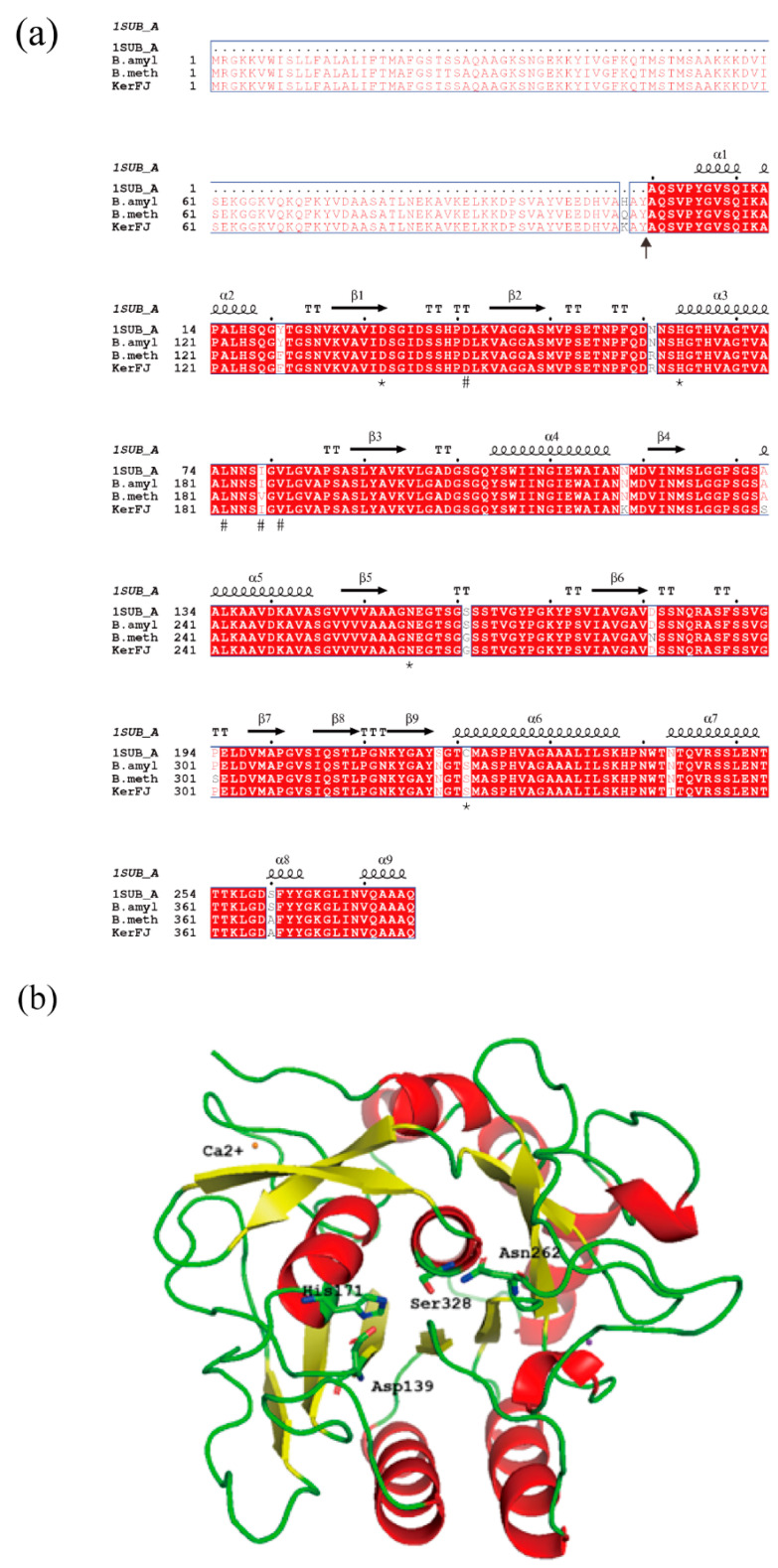
Sequence and structural analysis of keratinase KerFJ. (**a**) Multiple sequence alignment of the mature KerFJ protein with its closest homologs retrieved from the MEROPS peptidase database, including subtilisin BPN′ (1SUB_A), *Bacillus amyloliquefaciens* (*B. amyl*), and *Bacillus methylotrophicus* (*B. meth*). Conserved catalytic residues (Asp139, His171, and Ser328) are indicated by asterisks (*), while conserved substrate-binding residues are marked with hash symbols (#). Secondary structure elements (α-helices and β-strands) predicted from the 1SUB_A crystal structure are annotated above the alignment. (**b**) Predicted three-dimensional structure of mature KerFJ, generated using SWISS-MODEL with subtilisin BPN′ (PDB ID: 1SUB_A) as the template. The catalytic triad (Asp139, His171, Ser328) is shown as stick models, and a bound Ca^2+^ ion is depicted as an orange sphere. Structural elements are color-coded: α-helices (red), β-strands (yellow), and loops (green). The model exhibits a typical subtilisin-like fold, and the presence of Ca^2+^ supports the role of metal ions in stabilizing enzyme structure. No potassium ions were modeled.

Based on sequence alignment with the MEROPS database, the putative catalytic triad in KerFJ was Asp139, His171 and Ser328, as well as a conserved Asn262 (Figure 6a, marked by asterisks).

A three-dimensional structural model of KerFJ was generated using the SWISS-MODEL server, with PDB ID: 1SUB (chain A) as the template (Figure 6b). The model showed 96.7% sequence identity with the template and included a bound Ca^2+^ ion, likely contributing to structural stability. This is consistent with experimental results showing that chelation of divalent metal ions by EDTA and EGTA reduced enzyme activity, reinforcing the functional role of Ca^2+^ in maintaining enzymatic function.

## 4. Discussion

### 4.1. Production Optimization and Efficient Purification of KerFJ

Microbial keratinases offer a sustainable and eco-friendly solution for valorizing keratin-rich wastes such as feathers, wool, and hair. In this study, we identified, purified and for the first time comprehensively characterized a keratinase, KerFJ, produced by *Bacillus* sp. FJ-3-16—a strain taxonomically assigned to the *Bacillus* genus, which includes many species (e.g., *B. subtilis*, *B. licheniformis*) that are widely used in enzyme production and have been granted Generally Recognized as Safe (GRAS) status by the FDA [30]. These precedents highlight the recognized safety of *Bacillus*-derived enzymes for applications in food processing, animal feed, and related industries.

Fermentation medium composition plays a pivotal role in influencing microbial enzyme yields. In our previous study using statistical design methods, KerFJ production was significantly enhanced by using corn flour, soy flour, and casein as carbon and nitrogen sources, resulting in a yield of 166.08 U/mL after 48 h under the previous unit definition (0.1 absorbance increase min^−1^ = 1 U) [16]. In the present study, adopting the same optimized medium but extending the cultivation to 120 h and applying a different unit definition (0.01 absorbance increase min^−1^ = 1 U) led to an activity of 1800 U/mL, corresponding to a 22.5-fold increase compared to the unoptimized feather-based medium (80 U/mL). These findings highlight both the robustness of the optimized medium composition and the impact of cultivation duration and activity definition on reported enzyme yields. Similar observations have been reported in other keratinase-producing strains, where readily metabolizable substrates supported higher enzyme yields compared to raw keratin materials [31,32]. The use of low-cost, nutrient-rich agricultural byproducts like corn flour and soy flour offers clear industrial benefits, including faster biomass accumulation, higher enzyme yields, and reduced production costs. The 50 L bioreactor trial with peak KerFJ activity reached in just 18 h—much faster than flask culture—and a 26.5% higher yield [16], demonstrating the scalability and cost-effectiveness of *Bacillus* sp. FJ-3-16 as an industrial keratinase producer.

Purification of KerFJ was achieved using a straightforward two-step ion-exchange chromatography process. This strategy efficiently removed impurities with minimal activity loss, yielding a 25% recovery. The final purified preparation was highly concentrated, as evident by the strong band in SDS-PAGE and was obtained without the use of affinity tags or complex procedures, supporting its potential suitability for industrial enzyme production.

When comparing with other *Bacillus* keratinases, substantial variation in reported specific activities is observed, largely due to differences in the activity assay methods and substrates employed. For instance, *Bacillus* sp. RCM-SSR-102 keratinase, purified through ultrafiltration followed by Sephadex G-100 gel filtration (Cytiva, Marlborough, MA, USA) with azo-casein as substrate, exhibited a specific activity of 4848 U·mg^−1^ [33]; *Bacillus cereus* IIPK35 keratinase, purified via Sephadex G-75 gel filtration (Cytiva, Marlborough, MA, USA) with chicken feather as substrate, showed 808 U·mg^−1^ [12]; *Bacillus tequilensis* Q7 keratinase, purified using Mono S Sepharose cation-exchange chromatography (Cytiva, Marlborough, MA, USA) with keratin azure as substrate, reached 47,971 U·mg^−1^ [34]; and *Bacillus amyloliquefaciens* S13 keratinases KERZT-A and KERZT-B, purified by high-performance size exclusion chromatography (ZORBAX porous silica microspheres, PSM 300, Agilent, Beijing, China) with keratin azure as substrate, reached 50,526 U·mg^−1^ and 38,000 U·mg^−1^, respectively [26]. In comparison, the keratinase KerFJ in this study, purified via SP-Sepharose FF cation-exchange chromatography (Cytiva, Marlborough, MA, USA) with soluble keratin as substrate, exhibited a markedly higher specific activity of 53,000 U·mg^−1^. This variation likely reflects the influence of different assay substrates, reaction conditions, and detection methods on the apparent activity, as well as the ability of the final purification step to concentrate the active enzyme. The exceptionally high value for KerFJ may thus be attributed to both its intrinsic catalytic efficiency toward soluble keratin and the effective purification strategy, which preserved high activity while achieving high purity.

### 4.2. Enzymatic Characterization and Structural Analysis

Keratinases typically function under mild reaction temperatures (40–60 °C) and neutral to alkaline conditions (optimal pH 7.0–10.0) [17]. Similarly, KerFJ demonstrated optimal activity at 55 °C and retained over 85% activity within the 55–65 °C range, indicating good thermal adaptability. Although its half-life decreased at higher temperatures, KerFJ maintained a moderate thermostability (e.g., t_1_/_2_ = 10.6 h at 40 °C), which is sufficient for short-term industrial applications such as high-temperature washing or keratin pretreatment. In terms of pH performance, KerFJ exhibited peak activity at pH 9.5 and retained substantial catalytic efficiency across a broad alkaline range (pH 7.0–10.5). Notably, it remained highly stable even after pre-incubation at pH 12.0, demonstrating excellent alkaline resistance.

This combination of alkaline stability and moderate thermostability makes KerFJ particularly suitable for integration into industrial processes such as detergent formulations and leather treatment, where enzymes are often exposed to harsh alkaline and elevated temperature environments [35].

Substrate specificity assays revealed that KerFJ preferentially hydrolyzes unfolded or loosely packed protein substrates, as evidenced by its highest activity toward gelatin and casein. Although its activity on native keratin substrates such as feather powder and wool keratin was lower, it remained detectable. This suggests that KerFJ may be better suited for processing partially denatured keratin or in workflows that facilitate substrate unfolding.

Sequence alignment and MEROPS classification assigned KerFJ to the S8 family of subtilisin-like serine proteases, with a conserved catalytic triad (Asp139, His171, Ser328) and metal-binding residues consistent with Ca^2+^-dependent stability. Structural modeling based on subtilisin BPN′ confirmed the presence of a canonical subtilase fold, including the conserved catalytic triad and Ca^2+^-binding sites essential for structural integrity under alkaline conditions. This provides a structural basis for KerFJ’s alkaline and thermal tolerance, as Ca^2+^ binding is known to stabilize loop regions and maintain enzyme conformation under stress conditions [26,36,37].

Homology analysis revealed that KerFJ shared the highest sequence identity (97.9%, full length) with a putative keratinase from *Bacillus methylotrophicus* KJN2 (accession no. AGC81872.1), although its enzymatic properties have not been reported. The second highest identity (84.6%) was with a keratinase from *B. subtilis* S1-4 (QHN63939.1) [38], which also lacks application-related characterization. These findings, together with the experimentally validated catalytic performance of KerFJ, indicate that it represents a previously uncharacterized keratinase with potential utility in keratin waste biodegradation, leather processing, and detergent formulations.

Importantly, the conserved catalytic architecture, favorable native traits, and sequence divergence from other industrial Subtilisins support the hypothesis that KerFJ could serve as a promising scaffold for rational engineering or directed evolution aimed at further enhancing stability, substrate specificity, or detergent compatibility.

### 4.3. Hydrolysis Keratin Waste

The enzymatic degradation of keratin-rich waste such as feather, wool, and hair represents a sustainable alternative to harsh chemical or thermal hydrolysis methods, which often result in incomplete degradation or loss of functional amino acids [39,40]. In this study, KerFJ demonstrated substantial keratinolytic activity, with a feather degradation rate reaching 70% under mild reaction conditions (37 °C, 24 h, 2500 U/mL), highlighting its efficiency in converting recalcitrant keratin into soluble protein hydrolysates.

Compared with other reported keratinases, which often require higher temperatures or extended incubation times to achieve comparable degradation ratios [10,36,41], KerFJ achieved high feather degradation under relatively mild conditions, suggesting lower energy input and better suitability for industrial bioprocessing. Its moderate activity toward wool (40%) and human hair (15%) also indicates broader substrate flexibility and potential utility in processing a range of keratin wastes, especially when combined with pre-treatment strategies or enzyme cocktails.

The feather protein hydrolysate generated by KerFJ not only yielded a high soluble protein content (2.69 mg/mL; 38.4% recovery) but also exhibited a favorable amino acid composition. Notably, it was enriched in essential and hydrophobic amino acids such as phenylalanine, valine, methionine, alanine, and lysine. These amino acids are of nutritional importance and can serve as precursors for bioactive peptides [13,42].

In addition to nutritional value, the hydrolysate displayed significant antioxidant capacity, as evidenced by DPPH, ABTS, and FRAP assays. These results suggest that KerFJ facilitates both efficient keratin degradation and the generation of value-added hydrolysates enriched in bioactive amino acids and antioxidant activity, supporting its potential use in feed, supplements, or bio-based materials [33,43,44].

These findings collectively indicate that KerFJ can serve as an effective biocatalyst for the valorization of poultry feather waste—one of the largest sources of insoluble keratin globally. Its ability to simultaneously achieve high degradation efficiency and generate bioactive peptides under mild conditions supports its potential integration into circular biorefinery processes for keratinous biomass.

### 4.4. Dehairing

Keratinases have long been recognized as promising biocatalysts for sustainable depilation in both leather and meat industries, offering an eco-friendly alternative to traditional lime-sulfide methods that produce toxic effluents and often compromise hide quality [45]. However, most *Bacillus*-derived proteases—such as those from *B. subtilis*, *B. pumilus*, and *B. licheniformis*—have been evaluated mainly at laboratory scale, and often face challenges including prolonged reaction times, residual scud, or partial collagen degradation under suboptimal conditions [46,47].

In this study, KerFJ demonstrated robust keratinolytic activity and remarkable substrate compatibility, efficiently depilating pig, sheep, and donkey skins under mild conditions (45 °C, 8 h) while preserving collagen integrity, as confirmed by SEM analysis. To assess real-world applicability, factory-scale trials using ~20 kg of Bama Xiang pig skin, heads, and hooves revealed that KerFJ achieved complete or near-complete depilation within 7 h and retained >80% activity for reuse in a second batch, which reached full hair removal within 10 h. This performance demonstrates KerFJ’s scalability, efficiency, and cost-effectiveness, offering clear advantages over many previously reported keratinase systems that were constrained by limited scalability or prolonged reaction times.

Furthermore, KerFJ’s non-destructive action on donkey hides—a critical raw material for Ejiao (donkey-hide gelatin) production—demonstrates its potential for applications requiring preserved collagen structure. This feature aligns with previous reports of *Bacillus*-derived keratinases achieving efficient depilation without damaging the dermal matrix [21,35,46]. KerFJ matches or exceeds these precedents by effectively depilating structurally dense substrates such as donkey skin under mild conditions, with visual and microscopic evidence confirming intact collagen architecture. This high degree of selectivity and tissue preservation supports its application in value-added sectors such as traditional medicine and cosmeceutical processing, where gentle removal of hair is essential without compromising the structural or biochemical integrity of the hide.

From a food safety perspective, *Bacillus* species are widely used in the fermentation and enzyme industries, with certain strains designated as GRAS (Generally Recognized As Safe) by the FDA and other regulatory bodies for specific uses [30]. Overall, KerFJ’s ability to perform non-destructive, efficient depilation across diverse animal hides—combined with its GRAS-producing strain background—positions it as a promising candidate for large-scale, environmentally friendly leather and meat processing workflows.

### 4.5. Washing Performance

KerFJ demonstrated clear enhancement of stain removal efficacy when supplemented into a commercial detergent, particularly against protein-rich stains such as blood. This result is consistent with previous reports where other keratinases have shown visible improvement in removing blood stains [48,49]. In addition to visible improvement, this study combined quantitative reflectance analysis and SEM imaging, which confirmed the effective removal of blood residues and revealed smooth, residue-free fibers comparable to unstained controls. Notably, the fiber structure remained intact after treatment, indicating that KerFJ selectively hydrolyzes surface proteins without damaging the underlying textile matrix. In addition to blood, KerFJ also improved the removal of kitchen-related stains such as tomato sauce, chili paste, ginger powder, and soybean paste. These soils often contain complex mixtures of proteins, carbohydrates, and lipids. While keratinases are typically studied for their action on protein-rich substrates, the demonstrated efficacy of KerFJ on these mixed-composition stains highlights its broader cleaning potential.

KerFJ exhibited remarkable stability in the majority of tested commercial detergents, with some even enhancing its enzymatic activity after 12 h of incubation. This enhanced or retained activity—particularly notable in Liby (125.56% ± 1.58%), Ariel (114.11% ± 1.75%), and OMO (110.00% ± 3.19%)—suggests that certain detergent formulations may contain stabilizing agents that help preserve the enzyme’s structure or facilitate better substrate access. The fact that KerFJ retained ≥100% relative activity in more than half (6 out of 11) of the detergents tested underscores its broad compatibility and robustness under chemically complex conditions. This level of stability is superior to that reported for other keratinases such as those from *Bacillus zhangzhouensis* MH1 [49] and *Bacillus halotolerans* L2EN1 [50], which showed reduced activity in similar detergent matrices. Furthermore, although Tide and WhiteCat partially inhibited KerFJ activity, the inhibition levels were comparable to those observed in keratinases from *Bacillus cereus* YQ15 [51], indicating that KerFJ is not unusually sensitive to detergent-induced stress.

Compared to previously reported keratinases, KerFJ offers superior detergent stability and broader cleaning efficacy, highlighting its potential as a next-generation enzymatic additive for commercial laundry formulations targeting proteinaceous and complex stains.

## 5. Conclusions

In this study, an extracellular alkaline keratinase, KerFJ, was purified from *Bacillus* sp. FJ-3-16 and comprehensively characterized for the first time for its biochemical properties and industrial potential. The enzyme exhibited high production yield in low-cost optimized medium and was efficiently purified using a simple two-step ion-exchange chromatography. KerFJ demonstrated excellent activity and stability under alkaline conditions and moderate temperatures, as well as remarkable tolerance to surfactants, oxidants, and commercial detergent formulations.

KerFJ efficiently hydrolyzed recalcitrant keratin substrates such as feathers, wool, and human hair, generating hydrolysates enriched in amino acids and antioxidant peptides. The enzyme also enabled effective, non-destructive dehairing of animal hides including pig, sheep, and donkey skin, and enhanced the removal of protein-based stains from fabrics without damaging fibers.

Together, these results position KerFJ as a robust, multifunctional biocatalyst suitable for sustainable keratin waste valorization, eco-friendly leather processing, and enzymatic detergent applications. Its favorable native properties, combined with a safe production strain and broad application compatibility, make it a promising candidate for future industrial deployment and further protein engineering.

## Figures and Tables

**Table 1:** Summary of purification steps for keratinase KerFJ from *Bacillus* sp. FJ-3-16.

Purification Procedures	Activity (Units) × 10^3^	Total Protein (mg)	Activity Recovery Rate (%)	Specific Activity (U/mg of Protein)	Purification Factor (Fold)
Crude extract	8174	7712	100	1060	1
(NH_4_)_2_SO_4_ precipitation (40–70%)	5721.8	1817.48	70	3148.2	2.97
Q-Sepharose^TM^ FF	1798.28	56.55	22	31,800	30
SP-Sepharose^TM^ FF	2043.5	38.56	25	53,000	50

**Table 2:** Effects of protease inhibitors, reducing agents, oxidizing agents, and surfactants on the activity of keratinase KerFJ. Enzyme was pre-incubated with each compound at the indicated concentration in 50 mM Tris-HCl buffer (pH 8.0) at 25 °C for 20 min.

Category	Treatment	Residual Activity	Treatment	Residual Activity
	None	100.0 ± 0.65		
Inhibitors	PMSF (1 mM)	85.21 ± 3.51	PMSF (5 mM)	26.88 ± 1.29
	O-P (1 mM)	91.45 ± 2.91	O-P (5 mM)	88.93 ± 3.16
Chelators	EDTA (1 mM)	26.88 ± 1.65	EDTA (5 mM)	12.87 ± 2.26
	EGTA (1 mM)	15.81 ± 1.31	EGTA (5 mM)	13.51 ± 1.77
Reducing Agents (5 mM)	DTT	100.28 ± 4.32	DTNB	103.38 ± 4.10
	β-ME	112.26 ± 2.22		
Oxidizing Agents (3%)	H_2_O_2_	102.96 ± 0.93	NaClO	76.34 ± 3.12
Surfactants (1%)	Saponin	203.81 ± 7.49	Triton X-100	140.03 ± 6.55
	Tween-80	132.56 ± 3.44	Sodium cholate	87.29 ± 3.10
	SDS	30.81 ± 1.12		

Residual activity was expressed as a percentage of the control (set to 100%). Values represent mean ± standard deviation (*n* = 3).

**Table 3:** Categorized effects of metal ions (10 mM) on the activity of keratinase KerFJ. Enzyme was incubated with each metal ion in 50 mM Tris-HCl buffer (pH 8.0) at 25 °C for 20 min. Residual activity was calculated as a percentage relative to the control (100%). Metal ions were grouped based on their impact: enhancing (>105%), neutral (90–105%), and inhibitory (<90%).

Enhancing Ions	Residual Activity (%)	Neutral Ions	Residual Activity (%)	Inhibitory Ions	Residual Activity
Mn^2+^	137.71 ± 4.27	Mg^2+^	100.50 ± 3.03	Co^2+^	70.63 ± 2.46
K^+^	110.14 ± 2.00	Ca^2+^	99.30 ± 1.72	Fe^2+^	64.75 ± 3.48
Na^+^	109.03 ± 0.58	Ba^2+^	97.65 ± 1.41	Cu^2+^	58.11 ± 2.76
Sr^2+^	107.63 ± 3.88			Ni^2+^	54.21 ± 0.48
Li^+^	104.06 ± 4.34			Zn^2+^	28.67 ± 1.65

Data are presented as mean ± standard deviation (*n* = 3).

**Table 4:** Stability of keratinase KerFJ in the presence of commercial detergents (1% *v*/*v*). The enzyme was incubated with each detergent at 40 °C for 1, 6, and 12 h. Residual activity was determined under standard assay conditions and expressed as a percentage relative to the control (set to 100%). Based on the residual activity at 12 h, detergents were categorized as Enhanced (>110%), Stable (90–110%), or Inhibitory (<90%) based on their effect on enzyme activity.

Detergent (1%)	1 h (%)	6 h (%)	12 h (%)	Catagory
Liby	126.86 ± 4.44	126.23 ± 3.95	125.56 ± 1.58	Enhanced
Ariel	118.39 ± 0.77	116.02 ± 2.71	114.11 ± 1.75	
OMO	112.99 ± 3.91	112.63 ± 4.04	110.00 ± 3.19	
Bluemoon	112.87 ± 1.05	110.36 ± 2.22	102.82 ± 1.45	Stable
Amway	110.38 ± 1.57	108.59 ± 2.15	98.33 ± 3.33	
Kaimi	104.55 ± 1.13	110.03 ± 2.23	102.82 ± 1.45	
Supra	97.28 ± 1.52	86.34 ± 0.70	38.58 ± 0.97	Inhibitory
Whitecat	88.99 ± 3.01	45.55 ± 0.32	10.38 ± 0.29	
Diaopai	49.06 ± 0.30	14.15 ± 0.24	9.79 ± 0.19	
Tide	34.91 ± 1.84	12.69 ± 0.10	11.15 ± 0.57	
Control	100.00 ± 2.11	95.00 ± 0.11	85.00 ± 2.13	Reference Group

Data was presented as mean ± standard deviation (*n* = 3).

**Table 5:** Amino acid content in feather hydrolysate.

Amino Acid	Concentration (mg/mL)	Amino Acid	Concentration (mg/mL)
Asp	0.09 ± 0.01	Tyr	0.19 ± 0.02
Glu	0.43 ± 0.03	Cys	0.08 ± 0.01
Ser	0.03 ± 0.00	Val	1.85 ± 0.10
His	0.02 ± 0.00	Met	0.71 ± 0.04
Gly	0.03 ± 0.00	Phe	1.40 ± 0.08
Thr	0.09 ± 0.01	Ile	0.16 ± 0.01
Arg	0.08 ± 0.01	Leu	0.18 ± 0.01
Ala	0.48 ± 0.03	Lys	0.54 ± 0.02
Total	6.35 ± 0.22		

Data was presented as mean ± standard deviation (*n* = 3).

**Table 6:** Reflectance values (%) of stained cloths after different washing treatments.

Stain Type	Blank Control (Tap Water)	1% Ariel Detergent	1% Ariel + KerFJ	Reflectance Improvement (KerFJ vs. Ariel)
Ginger Powder	52.3 ± 0.8 ^a^	60.5 ± 1.1 ^b^	68.9 ± 0.9 ^c^	+8.4%
Tomato Sauce	47.6 ± 0.7 ^a^	58.1 ± 1.0 ^b^	69.2 ± 1.2 ^c^	+11.1%
Thick Chili Sauce	45.3 ± 0.9 ^a^	56.7 ± 1.3 ^b^	67.4 ± 1.0 ^c^	+10.7%
Sweet Soybean Paste	46.5 ± 0.6 ^a^	59.2 ± 1.2 ^b^	68.1 ± 0.8 ^c^	+8.9%
Blood	39.7 ± 1.0 ^a^	54.6 ± 1.5 ^b^	70.8 ± 1.3 ^c^	+16.2%

Note: Values represent the mean ± standard deviation (*n* = 3). Superscript letters (a, b, c) indicate significant differences between treatment groups within the same stain type, as determined by one-way ANOVA followed by Tukey’s multiple comparison test (*p* < 0.05).

## Data Availability

The 16S rRNA gene sequence of strain FJ-3-16 is available at NCBI under accession number MW677222.1. The full-length *kerFJ* gene sequence is available under accession number MZ516797.1. Other data are available from the corresponding author upon reasonable request.
